# Cerebral glucagon‐like peptide‐1 receptor activation alleviates traumatic brain injury by glymphatic system regulation in mice

**DOI:** 10.1111/cns.14308

**Published:** 2023-06-23

**Authors:** Chuanxiang Lv, Shuai Han, Zhuang Sha, Mingqi Liu, Shiying Dong, Chunyun Zhang, Zean Li, Kang Zhang, Shouyong Lu, Zhiyang Xu, Li Bie, Rongcai Jiang

**Affiliations:** ^1^ Department of Neurosurgery The First Hospital of Jilin University Changchun China; ^2^ Department of Neurosurgery Tianjin Medical University General Hospital Tianjin China; ^3^ Tianjin Neurological Institute, Key Laboratory of Post‐Neuroinjury Neuro‐repair and Regeneration in Central Nervous System Tianjin Medical University General Hospital, Ministry of Education Tianjin China

**Keywords:** cognitive impairment, glucagon‐like peptide‐1 receptor, glymphatic system, traumatic brain injury

## Abstract

**Aim:**

We aimed to assess the effects of cerebral glucagon‐like peptide‐1 receptor (GLP‐1R) activation on the glymphatic system and whether this effect was therapeutic for traumatic brain injury (TBI).

**Methods:**

Immunofluorescence was employed to evaluate glymphatic system function. The blood–brain barrier (BBB) permeability, microvascular basement membrane, and tight junction expression were assessed using Evans blue extravasation, immunofluorescence, and western blot. Immunohistochemistry was performed to assess axonal damage. Neuronal apoptosis was evaluated using Nissl staining, terminal deoxynucleotidyl transferase‐mediated dUTP nick end labeling (TUNEL) staining, and western blot. Cognitive function was assessed using behavioral tests.

**Results:**

Cerebral GLP‐1R activation restored glymphatic transport following TBI, alleviating BBB disruption and neuronal apoptosis, thereby improving cognitive function following TBI. Glymphatic function suppression by treatment using aquaporin 4 inhibitor TGN‐020 abolished the protective effect of the GLP‐1R agonist against cognitive impairment.

**Conclusion:**

Cerebral GLP‐1R activation can effectively ameliorate neuropathological changes and cognitive impairment following TBI; the underlying mechanism could involve the repair of the glymphatic system damaged by TBI.

## Graphical abstract

Cerebral GLP‐1R activation can effectively ameliorate neuropathological changes and cognitive impairment following traumatic brain injury, and the underlying mechanism could involve the repair of the glymphatic system damaged by traumatic brain injury.

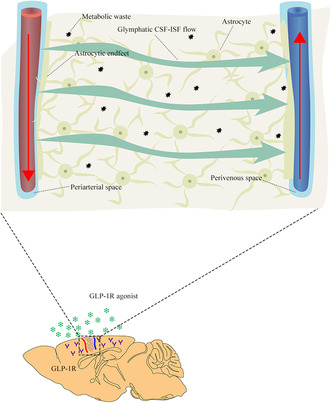

## 1 INTRODUCTION

Recent studies have demonstrated a brain‐wide network of paravascular pathways known as the glymphatic system, which utilizes perivascular channels formed by astroglial cells surrounding penetrating arteries and eliminates interstitial solutes and brain metabolic waste from the brain by facilitating fluid exchange between the cerebrospinal fluid (CSF) and interstitial fluid (ISF).^1^, ^2^, ^3^ Aquaporin 4 (AQP4), a water channel mainly localized to the astrocytes in a polarized manner surrounding the perivascular space, plays a significant role in glymphatic system regulation.^4^ Glymphatic dysfunction occurs when normal polarization of AQP4 is disrupted.^1^, ^3^, ^5^, ^6^ Amyloid beta and tau protein clearance from the interstitial spaces is significantly impaired in mice knocked out of AQP4 or treated by AQP4‐specific inhibitor TGN‐020.^3^, ^7^, ^8^ Several neurological diseases, such as Alzheimer's disease, Parkinson's disease, multiple sclerosis, and traumatic brain injury (TBI) are associated with glymphatic dysfunction attributed to perturbation of AQP4 expression or disruption of AQP4 polarization.^9^, ^10^

Traumatic brain injury is caused by external attacks, usually accompanied by serious neuropathological changes and cognitive impairment. Studies have demonstrated that the glymphatic system is severely damaged following TBI.^7^, ^11^ After TBI, glial fibrillary acidic protein (GFAP) positive astrocytes surround the ipsilateral hemisphere overproliferated and activated to form a consolidated glial scar, which alters the polarization and localization of AQP4. These disturbances following TBI lead to decreased fluid exchange and waste clearance in the glymphatic system, resulting in the accumulation of neurotoxic substances, such as amyloid beta and tau protein aggregates, which further increases the risk of developing neurodegenerative diseases.^7^, ^11^, ^12^, ^13^

Glucagon‐like peptide‐1 (GLP‐1) is an important peptide hormone, which possesses a variety of functions based on its receptor location, from regulating insulin secretion to controlling satiety and modulating autonomic nervous system activity.^14^, ^15^, ^16^ GLP‐1R in the brain is widely distributed in the cerebral cortex, hippocampus, hypothalamus, and brain stem. This widespread cerebral distribution of GLP‐1R confers GLP‐1 the potential to regulate multiple neurological and cognitive functions.^17^ However, after entering the extracellular space, endogenous GLP‐1 is quickly inactivated by dipeptidyl peptidase‐4 in a short time, which restricts its pharmacological use.^18^ Thus, Exendin‐4 (Ex‐4), an analog of GLP‐1 with structural modifications was synthesized. Emerging evidence from several studies has revealed that the activation of GLP‐1R signaling protects the brain against neuroinflammation, oxidative stress, and neurotoxicity in multiple disease models.^15^, ^18^, ^19^, ^20^ Thus, we hypothesized that cerebral GLP‐1R activation could ameliorate TBI‐induced neurological impairment by restoration of the glymphatic system.

## 2 MATERIALS AND METHODS

### 2.1 Animals

All the animal experimental procedures described in this study were approved by the Animal Care and Use Committee of Tianjin Medical University General Hospital, Tianjin, China; 8–12‐week‐old C57BL/6 male mice were purchased from HFK Bioscience Corporation, used in all the experiments.

### 2.2 Fluid percussion injury model

The fluid percussion injury (FPI) model was prepared as described previously.^21^ After isoflurane anesthesia, the mice were treated using an FPI device. The sham group underwent the same process except for the strike procedure.

### 2.3 Experiment design and drug administration

**FIGURE 1:**
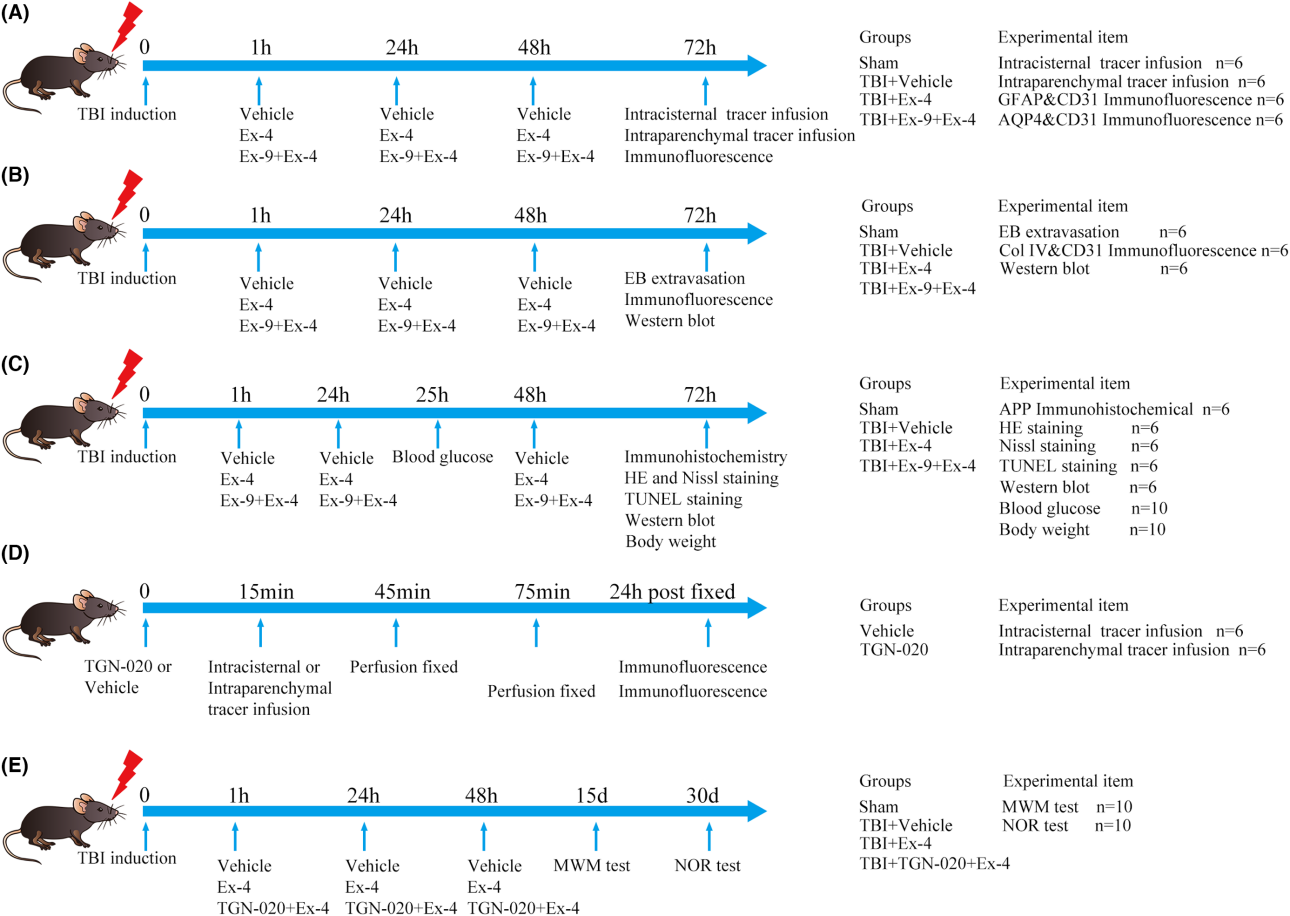
Basic characteristics of the study. (A) Experiment 1. Effects of cerebral GLP‐1R activation on the glymphatic system 3 days following TBI. (B) Experiment 2. Effects of cerebral GLP‐1R activation on the BBB 3 days following TBI. (C) Experiment 3. Effects of cerebral GLP‐1R activation on axonal injury and neuronal apoptosis 3 days following TBI. (D) Experiment 4–1. Effects of TGN‐020 on glymphatic transport function. (E) Experiment 4–2. Effects of cerebral GLP‐1R activation on cognitive function following TBI. EB, Evans blue; Ex‐4, Exendin‐4; Ex‐9, Exendin‐(9‐39); GLP‐1R, glucagon‐like peptide‐1 receptor; MWM test, Morris water maze test; NOR test, novel object recognition test; TBI, traumatic brain injury; TUNEL staining, terminal deoxynucleotidyl transferase‐mediated dUTP nick end labeling staining.

The animals were randomly assigned to four separate experiments (Figure 1). Ex‐4 (HY‐13443, MedChemExpress), a GLP‐1R agonist, was dissolved in physiological saline and delivered (10 μg/kg, intravenously) at 1 h following TBI. The GLP‐1R competitive antagonist Exendin‐(9‐39) (Ex‐9, HY‐P0264, MedChemExpress) was dissolved in physiological saline and was delivered (50 μg/kg, intravenously) 20 min before Ex‐4 administration. TGN‐020 (HY‐W008574, MedChemExpress), a specific inhibitor of AQP4, which was used as an effective means for pharmacological blocking of the glymphatic system, was dissolved in a cyclodextrin derivative, 20% sodium sulphobutylether‐β‐cyclodextrin (C871854, Macklin) in water for injection as described previously.^8^ TGN‐020 was delivered (250 mg/kg, intraperitoneally) 15 min before Ex‐4 administration.

### 2.4 Intracisternal tracer infusions

A fluorescent CSF tracer (rhodamine B isothiocyanate‐dextran; R9379, Sigma Aldrich, 70 KDa, RITC‐dextran) was dissolved in artificial CSF at a concentration of 0.5%. First, the mice were anesthetized and fixed in a stereotactic frame, followed by surgical exposure of the cisterna magna. The CSF tracer was subsequently infused into the subarachnoid CSF via cisterna magna puncture at a rate of 1 μL/min for 10 min (10 μL total volume) through a syringe pump (KDS LEGATO 130, RWD Life Science). Thereafter, the anesthetized mice were transcardially perfused and fixed with 4% paraformaldehyde (PFA) 30 min following the start of the infusion. The brains were subsequently dissected and post‐fixed in 4% PFA for 24 h before being sliced into 100‐μm coronal sections. Tracer influx into the brain was imaged ex vivo using whole‐slice conventional fluorescence microscopy (Olympus BX61) and quantified using ImageJ software (version 1.53, National Institutes of Health) as described previously.^7^

### 2.5 Intraparenchymal injections

To evaluate the interstitial metabolite clearance pathway, a fluorescent CSF tracer was stereotactically microinjected into the cerebral cortex of the mice. A 33‐gauge stainless steel cannula (Hamilton) was inserted at the following stereotactic coordinates within the cerebral cortex: 2.00 mm posterior, 1.50 mm lateral to the bregma, and 2.00 mm below the brain surface. 30 min following the cannula insertion, 500 nL of the CSF tracer was infused over 10 min. One hour later, the animal was rapidly perfusion fixed. Tracer efflux was quantified as described above.

### 2.6 AQP4 polarization evaluation

AQP4 polarization was evaluated 3 days following TBI as described previously.^5^, ^8^ Briefly, the median immunofluorescence intensity of the perivascular regions was measured. Thereafter a threshold analysis measured the percentage of the region exhibiting AQP4 immunofluorescence greater than or equal to perivascular AQP4 immunofluorescence (AQP4 percentage area). Polarization was expressed as the percentage of the region that exhibited lower AQP4 immunoreactivity than the perivascular endfeet (polarization = 100−AQP4 percentage area).

### 2.7 Evans blue extravasation

Evans blue (EB) extravasation was used to assess BBB permeability as described previously.^22^ Briefly, EB solution (4 mL/kg, 2% in saline; E2129, Sigma Aldrich) was administered via the tail vein and circulated for 2 h. After incubation with methylamide, the samples were centrifuged. Supernatant absorbance was measured using a spectrofluorophotometer to quantify EB concentration.

### 2.8 HE and Nissl staining

Mouse brain tissue was fixed with 4% PFA, followed by dehydration in series concentrations of ethanol. The paraffin‐embedded brain tissues were subsequently cut into 8‐μm slices for HE (G1120, Solarbio) and Nissl (G1432, Solarbio) staining. HE and Nissl staining were performed as described previously.^23^ The sections were observed under a light microscope (IX73, Olympus Corporation).

### 2.9 Immunohistochemical staining

Immunohistochemistry (IHC) was used to detect amyloid precursor protein (APP) as previously described.^24^ After incubation in 0.3% H_2_O_2_ for 30 min, the antigen was retrieved by boiling the sections in citrate buffer. Thereafter, 1% bovine serum was added to the sections for 30 min. The sections were incubated with primary rabbit anti‐APP antibody (1:500, ab32136, Abcam) overnight at 4°C. Secondary biotinylated goat anti‐rabbit antibody (GK500705, Gene Tech) was subsequently applied for 1 h at room temperature. The sections were observed under a light microscope.

### 2.10 Immunofluorescence

Immunofluorescence analysis was performed as described previously.^7^ The tissue sections were incubated with the following primary antibodies: AQP4 (1:500, 59678, Cell Signaling Technology), GFAP (1:500, 3670, Cell Signaling Technology), CD31 (1:500, AF3628, R&D Systems), NeuN (1:500, ab177487, Abcam), and collagen IV (1:500, ab19808, Abcam). The slices were subsequently incubated with species‐specific fluorescence‐conjugated secondary antibodies. Finally, the sections were observed under a fluorescence microscope and assessed using ImageJ software.

### 2.11 Western blot

Western blot was performed as previously described.^25^ A protein sample was collected from the TBI area of the brain tissue. The polyvinylidene difluoride membranes (PVDF, Millipore) were blocked with 5% skim milk and incubated at 4°C overnight with the following primary antibodies: ZO‐1 (1:1000, 61‐7300, ThermoFisher Scientific), claudin‐5 (1:1000, 35‐2500, ThermoFisher Scientific), occludin (1:1000, 27260‐1‐AP, Proteintech), caspase‐3 (1:1000, A0214, ABclonal), Bcl2 (1:1000, A0208, ABclonal), and β‐actin (1:1000, TA‐09, ZSGB‐BIO) antibodies. The membranes were subsequently incubated with secondary antibodies for 1 h. The blots were obtained with a ChemiDoc Touch Imaging System and quantified using ImageJ software.

### 2.12 TUNEL staining

Apoptotic cells were detected by TUNEL assay using the TUNEL assay kit (G3250, Promega) according to the manufacturer's instructions. Cortical neurons were labeled using NeuN immunofluorescence staining. The percentage of TUNEL‐positive neurons was quantified using a fluorescence microscope and ImageJ software.

### 2.13 Behavioral tests

The Morris water maze (MWM) test was conducted based on a previous protocol to test the spatial learning and memory of the mice.^24^ The novel object recognition (NOR) test was performed to assess short‐term memory 30 days following TBI, according to previous methods.^26^, ^27^

### 2.14 Statistical analysis

All the statistical analyses were performed using SPSS 26.0 software (IBM Corporation). Data are presented as mean ± standard deviation (SD). The Shapiro–Wilk test was used to assess the normality of the data distribution. Statistical differences among the two groups were analyzed by unpaired two‐tailed Student's *t*‐test. One‐way or two‐way analysis of variance (ANOVA) with Tukey's post hoc test was performed for comparison among multiple groups. Statistical significance was set at *p* < 0.05.

## 3 RESULTS

### 3.1 Cerebral GLP‐1R activation treatment attenuated glymphatic system dysfunction 3 days following TBI

**FIGURE 2:**
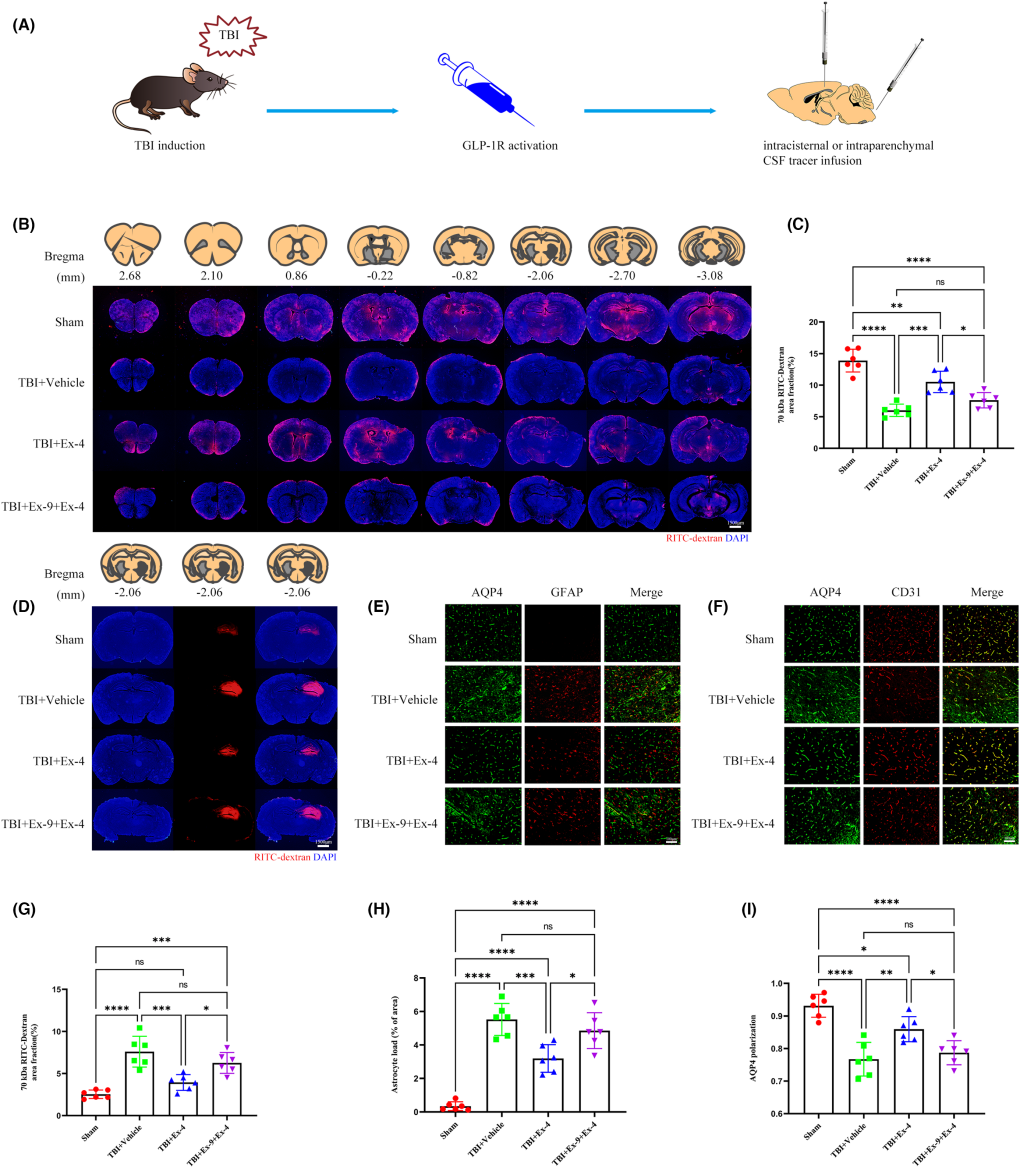
Cerebral GLP‐1R activation treatment attenuated glymphatic system dysfunction 3 days following TBI. (A) schematic shows that fluorescent tracer was injected into the cisterna magna or brain parenchyma after cerebral GLP‐1R activation treatment 3 days following TBI. (B) Representative brain sections stained for nuclei (DAPI; blue) and RITC‐dextran (red) influx into the brain parenchyma of mice from four groups. Scale bar, 1500 μm. (C) Quantification of the percentage of RITC‐dextran covered area fraction in brain sections in (B). *n* = 6 mice per group. (D) Representative brain sections stained for nuclei (DAPI; blue) and RITC‐dextran (red) clearance from the brain parenchyma of mice from four groups. Scale bar, 1500 μm. (E) Coimmunofluorescence staining for AQP4 (green) and GFAP (red) around the lesion. Scale bar, 100 μm. (F) Coimmunofluorescence staining for AQP4 (green) and CD31 (red) around the lesion. Scale bar, 100 μm. (G) Quantification of the percentage of residual RITC‐dextran covered area fraction in brain sections in (D). *n* = 6 mice per group. (H) Quantification of the percentage of GFAP covered area fraction in brain sections. *n* = 6 mice per group. (I) Quantification of the AQP4 polarization of mice from four groups. *n* = 6 mice per group. All data are shown as mean ± SD. *p < 0.05, **p < 0.01, ***p < 0.001, ****p < 0.0001. AQP4, aquaporin 4; Ex‐4, Exendin‐4; Ex‐9, Exendin‐(9‐39); GFAP, glial fibrillary acidic protein; GLP‐1R, glucagon‐like peptide‐1 receptor; RITC‐dextran, rhodamine B isothiocyanate‐dextran; TBI, traumatic brain injury.

Glymphatic CSF‐ISF exchange has been demonstrated to play a central role in waste clearance.^28^ However, this fluid exchange function is severely compromised following TBI.^7^ To evaluate the therapeutic effect of cerebral GLP‐1R activation on the paravascular CSF penetration following TBI, 10 μL of fluorescent CSF tracer was cautiously injected into the subarachnoid CSF of the cisterna magna. 30 min after injection, the brains were perfusion fixed. CSF tracer penetration into the brain parenchyma was evaluated using a conventional fluorescence microscope. Compared with sham mice, CSF tracer penetration into the brain with TBI was significantly decreased. Ex‐4 treatment significantly increased perivascular influx of the CSF tracer. However, when Ex‐9 was administered for 20 min beforehand, improvement in CSF penetration attributed to Ex‐4 was not observed (Figure 2A–C).

To evaluate the therapeutic effect of cerebral GLP‐1R activation on interstitial solute clearance post‐TBI, 0.5 μL of fluorescent CSF tracer was stereotactically microinjected into the parenchymal cortex around the lesion. One hour after injection, the brains were perfusion fixed. The function of interstitial solute clearance was measured by conventional fluorescence microscopy, comparing the residual amount of fluorescent tracer in the brain tissue of mice in each group; the residual fluorescent tracer in the brain tissue with TBI was significantly increased and Ex‐4 treatment effectively reduced the fluorescent tracer residue. When Ex‐9 was administered previously, improvement in the interstitial solute clearance induced by Ex‐4 disappeared (Figure 2A,D,G).

Previous studies have demonstrated that paravascular CSF recirculation and ISF solute clearance are dependent upon the polarized astroglial AQP4 water channel, which facilitates fluid movement between the perivascular and interstitial spaces.^5^, ^28^ Widespread reactive astrogliosis post‐TBI is closely associated with the loss of perivascular AQP4 polarization.^5^, ^7^ First, we evaluated reactive astrogliosis 3 days following TBI by immunofluorescence staining for GFAP and AQP4. GFAP expression was significantly higher in the TBI + vehicle group than that in the sham group. After Ex‐4 treatment, GFAP expression was reduced. However, when Ex‐9 was administered beforehand, GFAP expression remained high even following Ex‐4 treatment (Figure 2E,H). We subsequently evaluated the AQP4 polarization by immunofluorescence staining of CD31 and AQP4. Consistent with previous results, AQP4 expression in the perivascular extremity was significantly reduced 3 days following TBI. AQP4 polarization was effectively restored by Ex‐4 treatment. However, when Ex‐9 was administered 20 min beforehand, Ex‐4 treatment did not exert a protective effect against AQP4 polarization (Figure 2F,I).

### 3.2 Cerebral GLP‐1R activation treatment attenuated BBB disruption 3 days following TBI

**FIGURE 3:**
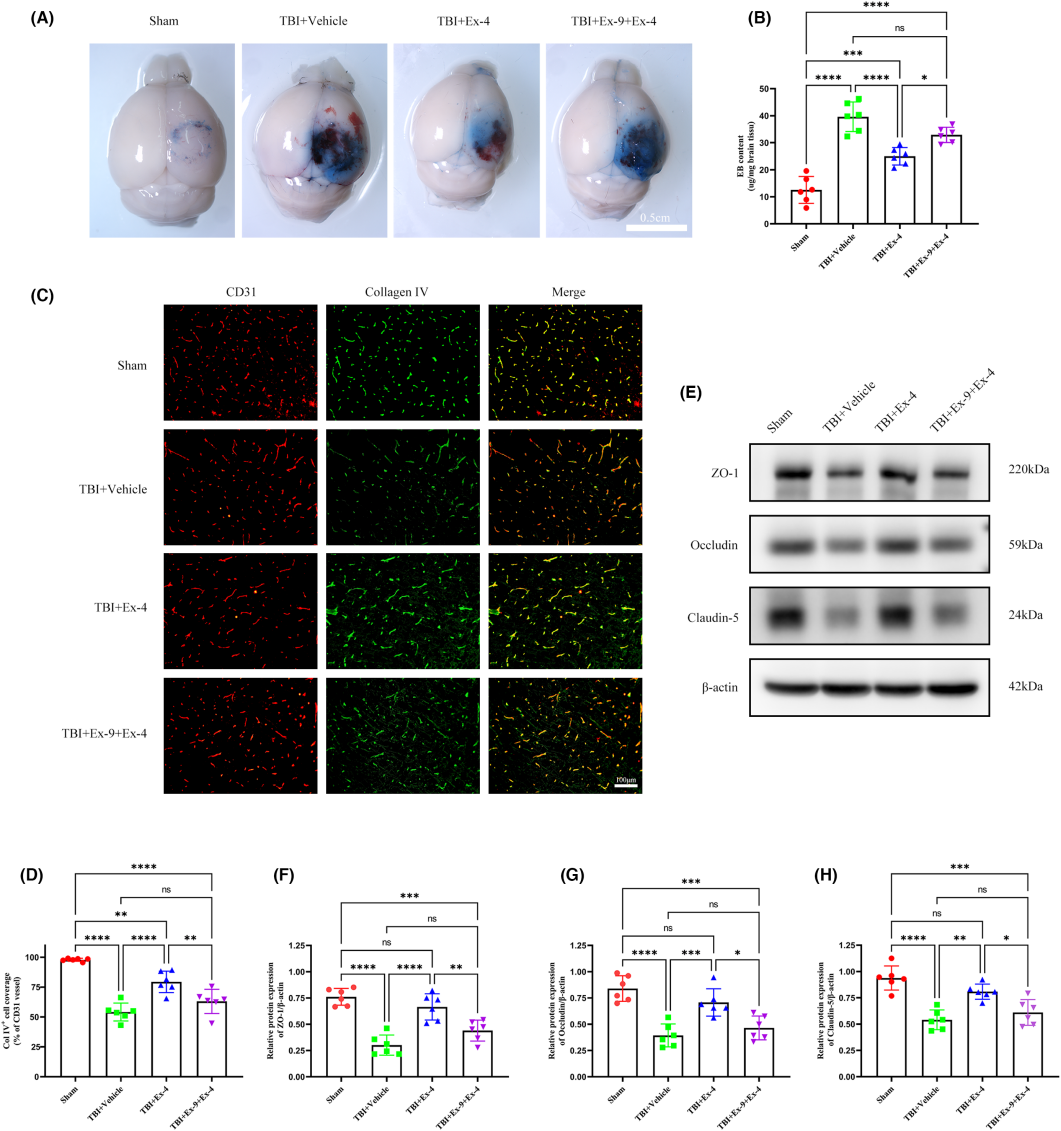
Cerebral GLP‐1R activation treatment attenuated BBB disruption 3 days following TBI. (A) Representative images of the EB extravasation assay. Scale bar, 0.5 cm (B) Quantification of EB concentration for each group. *n* = 6 mice per group. (C) Coimmunofluorescence staining for Collagen IV (green) and CD31 (red) around the lesion. Scale bar, 100 μm. (D) Quantification of Collagen IV + basement membrane coverage on CD31 microvessels. *n* = 6 mice per group. (E) Representative Western blots of ZO‐1, occludin and claudin‐5. (F–H) Quantification of relative protein expression normalized to the optical density of β‐actin. *n* = 6 mice per group. All data are shown as mean ± SD. *p < 0.05, **p < 0.01, ***p < 0.001, ****p < 0.0001. BBB, blood–brain barrier; Col IV, Collagen IV; EB, Evans blue; Ex‐4, Exendin‐4; Ex‐9, Exendin‐(9‐39); GLP‐1R, glucagon‐like peptide‐1 receptor; TBI, traumatic brain injury.

Similar to the glymphatic system, the BBB plays an important role in brain fluid exchange and substance clearance, which is regulated by AQP4. Thus, we examined the effect of cerebral GLP‐1R activation on the BBB function. EB extravasation was employed to assess BBB permeability. Three days following TBI, we observed that errhysis and EB dye extravasation in the ipsilateral hemisphere in the TBI group were significantly increased compared to those in the sham group, indicating severe BBB breakdown. However, the increased EB levels and errhysis were markedly attenuated by EX‐4 treatment. No obvious discrepancies in the EB levels or errhysis were observed between the TBI + vehicle and TBI + Ex‐9 + Ex‐4 groups (Figure 3A,B). Collagen IV and CD31 immunofluorescence staining was performed to detect the integrity of the basement membrane of the microvessels as described previously.^29^ Collagen IV basement membrane coverage of CD31 microvessels decreased significantly 3 days following TBI. Cerebral GLP‐1R activation effectively alleviated damage to the microvessel basement membrane (Figure 3C,D). Tight junction proteins are essential for maintaining the BBB. Therefore, tight junction protein (ZO‐1, occludin, and claudin‐5) expression was quantified using Western blot 3 days following TBI. After TBI, GLP‐1R activation effectively reversed the reduction and largely maintained the protein levels of ZO‐1, occludin, and claudin‐5 (Figure 3E–H). Overall, these results indicate that TBI‐induced BBB disruption can be salvaged by cerebral GLP‐1R activation.

### 3.3 Cerebral GLP‐1R activation treatment attenuated axonal injury and neuronal apoptosis 3 days following TBI

**FIGURE 4:**
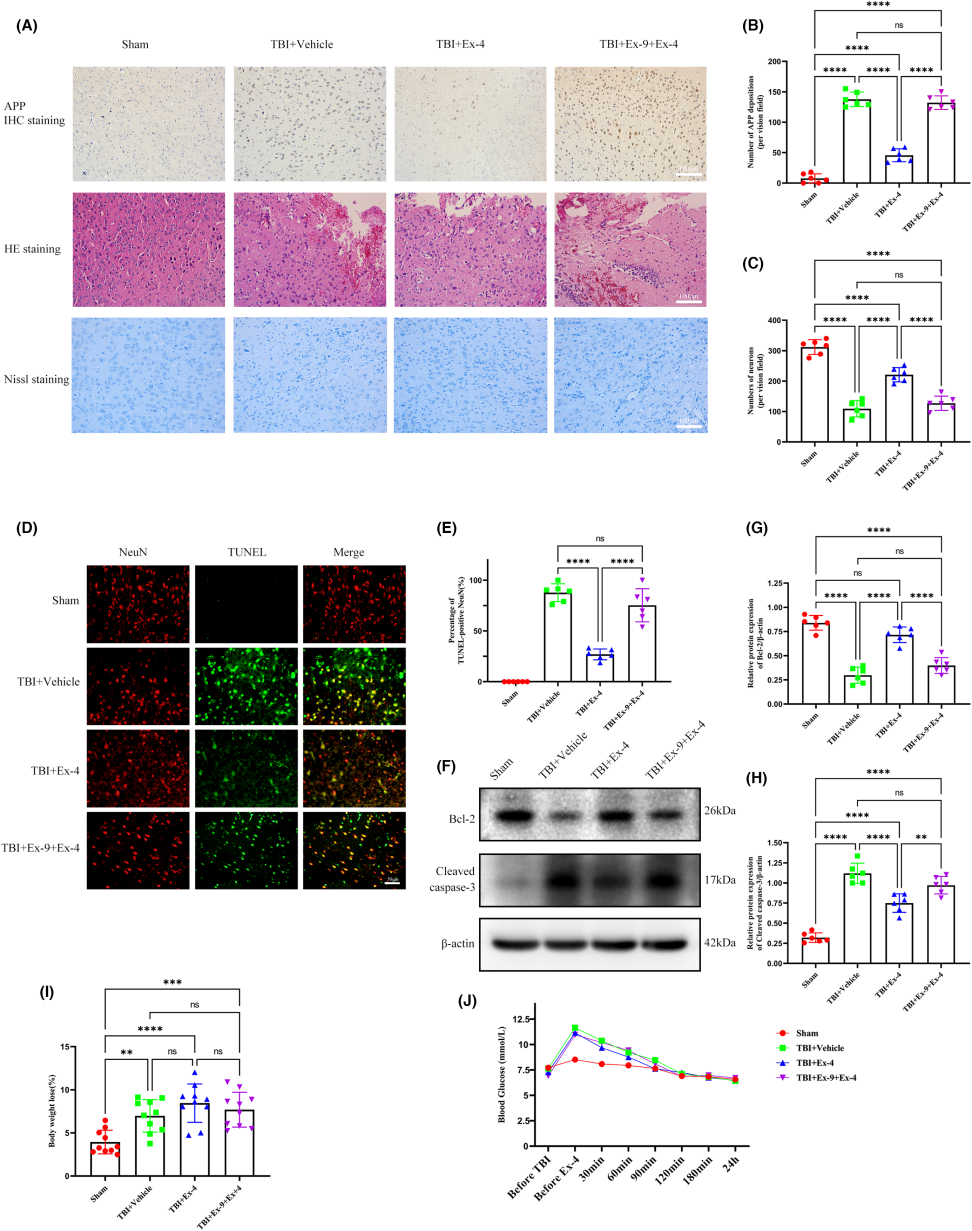
Cerebral GLP‐1R activation treatment attenuated axonal injury and neuronal apoptosis 3 days following TBI. (A) The representative images of APP immunohistochemistry, H&E and Nissl staining of ipsilateral cerebral cortex 3 days following TBI from four groups. Scale bar, 100 μm. (B) Quantitative analysis of APP accumulation. *n* = 6 mice per group. (C) Quantification of Nissl staining for neuronal loss analysis at 3 days following TBI. *n* = 6 mice per group. (D) TUNEL assay (green) and costaining of NeuN (red) in injured cortex. TUNEL^+^/NeuN^+^ cells are apoptotic neurons. Scale bar, 50 μm. (E) Count of apoptotic neurons in the cortex for each group. *n* = 6 mice per group. (F) Representative Western blots of cleaved caspase‐3, and Bcl‐2. (G, H) Quantification of relative protein expression normalized to the optical density of β‐actin. *n* = 6 mice per group. (I) The effects of cerebral GLP‐1R activation on body weight loss at 3 days following TBI. *n* = 10 mice per group (J) The effects of cerebral GLP‐1R activation on blood glucose change at 24 h following Ex‐4 administration. *n* = 10 mice per group. All data are shown as mean ± SD. *p < 0.05, **p < 0.01, ***p < 0.001, ****p < 0.0001. APP, amyloid precursor protein; Ex‐4, Exendin‐4; Ex‐9, Exendin‐(9‐39); GLP‐1R, glucagon‐like peptide‐1 receptor; HE staining, Hematoxylin and Eosin staining; IHC staining, Immunohistochemistry staining; TBI, traumatic brain injury; TUNEL (staining), terminal deoxynucleotidyl transferase‐mediated dUTP nick end labeling (staining).

Previous studies have demonstrated that axonal injury following TBI increases metabolic waste products in the brain, resulting in an increased burden on the glymphatic system.^7^ Immunohistochemical staining with an anti‐APP antibody, which has previously been shown to accumulate in injured axons, was performed 3 days following TBI.^24^ Representative images demonstrated that TBI caused a significant increase in the APP accumulation compared to that in sham mice. Cerebral GLP‐1R activation effectively alleviated APP deposition in the cortex around the lesion (Figure 4A,B). We subsequently determined the neuroprotective effect of GLP‐1R activation on TBI. HE staining was performed to evaluate the histological morphology of the cerebral cortex. As shown in the representative picture (Figure 4A), Ex‐4 administration significantly alleviated TBI‐induced cerebral cortex damage and loss as well as traumatic cerebral parenchymal hemorrhage. However, this protective effect was not observed in the TBI + Ex‐9 + Ex‐4 group. To assess the influence of GLP‐1R activation on neuronal death and degeneration following TBI, we performed TUNEL and Nissl staining of the damaged brain tissue of TBI mice 3 days following injury. As indicated by the representative images (Figure 4A,C,D,E), cerebral GLP‐1R activation effectively controlled TBI‐induced neuronal degeneration and apoptosis, thereby increasing the survival of neurons 3 days following TBI. Consistent with the staining assays described above, cerebral GLP‐1R activation following TBI upregulated Bcl‐2 expression and downregulated cleaved caspase‐3 expression in the cortex of the TBI group, as shown in the western blot analysis (Figure 4F–H).

The distributed dispersion of GLP‐1R increases the complexity of its function. Weight loss and hypoglycemia associated with Ex‐4 should be considered during TBI treatment. Body weight loss in all the groups was calculated using the body weight before surgery and 3 days following TBI. The body weight of each group decreased to varying degrees, with no significant difference in the weight loss between the TBI groups (Figure 4I). Changes in the blood glucose levels were monitored until 24 h following Ex‐4 administration. The blood glucose levels in each group significantly increased owing to the stress response to surgery, with no significant differences among the TBI groups (Figure 4J).

### 3.4 Cerebral GLP‐1R activation treatment can effectively alleviate cognitive impairment following TBI, which can be reversed by pharmacological blocking of the glymphatic system

**FIGURE 5:**
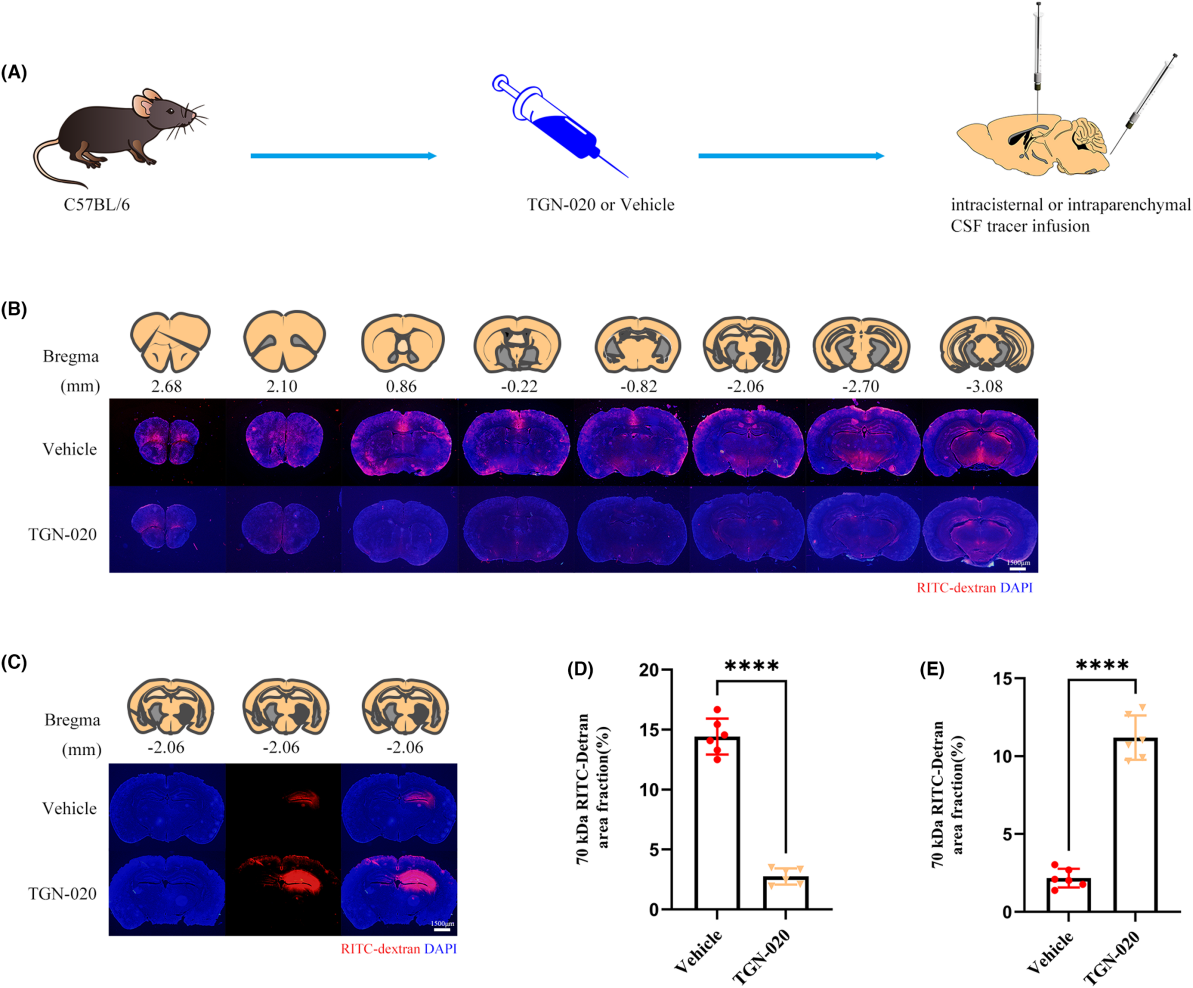
The glymphatic transport is pharmacological blocked by TGN‐020 (A) schematic shows that fluorescent tracer was injected into the cisterna magna or brain parenchyma after TGN‐020 or vehicle (cyclodextrin derivative) treatment. (B) Representative brain sections stained for nuclei (DAPI; blue) and RITC‐dextran (red) influx into the brain parenchyma of mice from two groups. Scale bar, 1500 μm. (C) Representative brain sections stained for nuclei (DAPI; blue) and RITC‐dextran (red) clearance from the brain parenchyma of mice from two groups. Scale bar, 1500 μm. (D) Quantification of the percentage of RITC‐dextran covered area fraction in brain sections in (B). *n* = 6 mice per group. (E) Quantification of the percentage of residual RITC‐dextran covered area fraction in brain sections in (C). *n* = 6 mice per group. All data are shown as mean ± SD. *p < 0.05, **p < 0.01, ***p < 0.001, ****p < 0.0001. CSF, cerebrospinal fluid; RITC‐dextran, rhodamine B isothiocyanate‐dextran.

Previously, it was reported that astrocytic AQP4 polarization could be interrupted by TGN‐020. Therefore, TGN‐020 can be used for effective pharmacological blocking of the glymphatic system.^8^ Prior to its blocking effect, we examined its effect on the glymphatic transport of RITC‐dextran. As shown in the representative images (Figure 5A–E), the glymphatic transport of RITC‐dextran was effectively blocked by TGN‐020.

**FIGURE 6:**
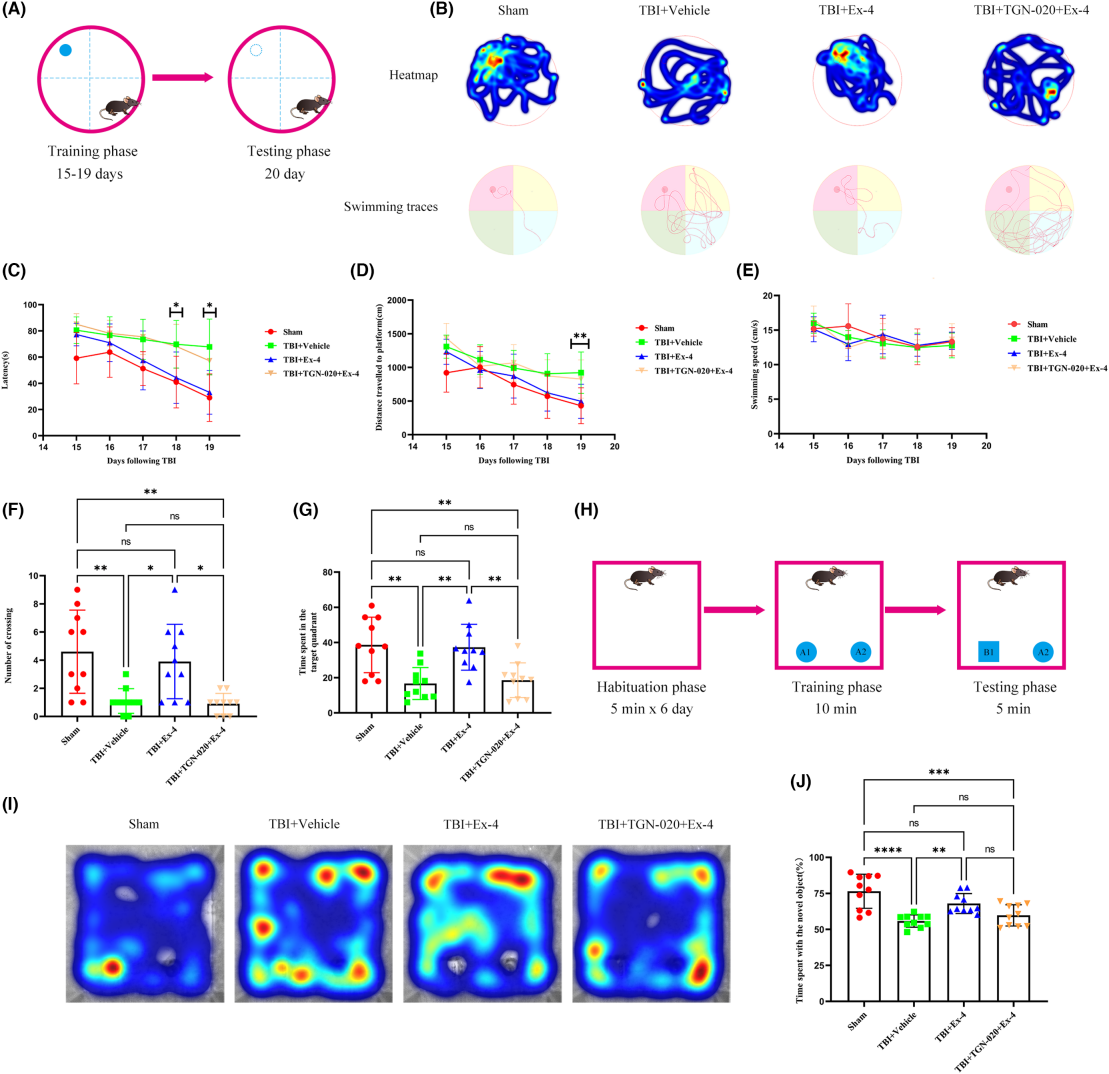
Cerebral GLP‐1R activation treatment can effectively alleviate cognitive impairment following TBI, which can be reversed by pharmacological blocking of glymphatic system. (A) Schematic representation of the method and process for the MWM test. (B) Representative thermal imaging of the probe trial and swimming traces for the MWM test. (C–G) Data from the MWM test was analyzed to evaluate the spatial learning and memory ability of mice following TBI. *n* = 10 mice per group (H) Schematic representation of the method and process for the NOR test. (I) Representative thermal imaging of the probe trial for the NOR test. (J) Time spent with the novel object was analyzed to evaluate the memory ability of mice following TBI. *n* = 10 mice per group. All data are shown as mean ± SD. *p < 0.05, **p < 0.01, ***p < 0.001, ****p < 0.0001. Ex‐4, Exendin‐4; Ex‐9, Exendin‐(9‐39); GLP‐1R, glucagon‐like peptide‐1 receptor; MWM test, Morris water maze test; NOR test, novel object recognition test; TBI, traumatic brain injury.

To study the effect of cerebral GLP‐1R activation on impaired cognitive function following TBI, we performed the MWM and NOR tests. In the MWM test, the escape latency of the TBI + vehicle group was greater than that of the sham group, and Ex‐4 administration decreased escape latency compared to the TBI + vehicle group on day 18 and day 19 following TBI. When TGN‐020 was administered 15 min beforehand, Ex‐4 administration failed to decrease escape latency (Figure 6A–C). The distance traveled to the platform was shorter in the TBI + Ex‐4 group than in the TBI + Vehicle group on day 19 (Figure 6D). No significant difference was observed in the swimming speed between all the groups (Figure 6E). Thereafter, we removed the hidden platform on day 20 following TBI to evaluate the number of crossings at the platform site, and a significant increase in the crossing number was observed in the TBI + Ex‐4 group compared to that in the TBI + vehicle group. When TGN‐020 was administered beforehand, no improvement was observed in the number of crossings treated with Ex‐4 (Figure 6F). Furthermore, the TBI + vehicle group spent less time in the target region than the sham group, and Ex‐4 treatment ameliorated this phenomenon. No significant differences were observed between the TBI + vehicle and TBI + TGN‐020 + Ex‐4 groups (Figure 6G). In the NOR test, the TBI + vehicle group spent a lower percentage of time around the novel object than the sham group. Ex‐4 treatment significantly increased the percentage of time around the novel object. TGN‐020 administration before Ex‐4 slightly counterbalanced this improvement, though not statistically significantly (Figure 6H–J). Collectively, these data demonstrated that Ex‐4 treatment has a neuroprotective effect on the neurological function following TBI, which can be reversed by TGN‐020 administered beforehand.

## 4 DISCUSSION

Previous studies have indicated that the glymphatic system is closely related to a variety of central nervous system diseases.^9^, ^28^ The glymphatic influx and solute clearance function were seriously impaired following TBI, resulting in the accumulation of neurotoxic substances, such as amyloid beta and tau protein in the brain.^7^

GLP‐1 was originally identified as a 31‐amino acid polypeptide secreted by L cells in the small intestine. It is also produced in the brain and plays neuroprotective roles by activating the GLP‐1 receptor.^18^ Studies have demonstrated that the number of amyloid beta and dense‐core plaques in the cortex of Alzheimer's mice model APP/PS1 mice treated with liraglutide (GLP‐1R agonist) decreased by 40%–50%, while the level of soluble amyloid oligomers decreased by 25%.^30^ Our results demonstrated that cerebral GLP‐1R activation significantly increased paravascular fluid exchange and attenuated glymphatic system destruction post‐TBI.

Studies have demonstrated that mild‐to‐moderate reactive astrogliosis and the accompanying loss of perivascular AQP4 polarization are important factors attributed to glymphatic pathway destruction following TBI.^7^ In previous studies, Ex‐4 was able to lower amyloid beta (1–42) induced oxidative stress and inflammation in astrocytes, demonstrating excellent neuroprotective effects.^18^ This could promote a reasonable hypothesis for cerebral GLP‐1R activation to improve the function of glymphatic system. Our results demonstrate that cerebral GLP‐1R activation can effectively ameliorate the reactive astrogliosis and loss of perivascular AQP4 polarization following TBI, thereby improving the transport function of glymphatic system.

In addition to the glymphatic pathway, the BBB also plays an important role in facilitating the transmission of neuroactive agents to maintain homeostasis of the brain microenvironment.^31^ BBB destruction, which occurs within hours or days following injury, is closely related to edema, neuroinflammation, and cell death.^32^ We demonstrated that cerebral GLP‐1R activation ameliorated TBI‐induced decrease of tight junction protein and microvessel coverage of collagen IV basement membrane, protecting the integrity and function of the BBB.

Traumatic brain injury is an established risk factor for progressive neurodegenerative diseases. Following TBI‐induced axonal injury, high levels of tau are released into the interstitial fluid. Axonal injury following TBI is considered to play a central role in the generation of proteinopathies of hyperphosphorylated tau and amyloid beta. A considerable portion of interstitial monomeric tau is cleared from the cortex by the glymphatic system.^7^, ^33^ APP immunohistochemistry demonstrated that cerebral GLP‐1R activation effectively alleviated the axonal injury caused by TBI, thereby reducing glymphatic system burden. Apoptosis is considered a key mechanism of cell death following TBI.^34^ Our results demonstrated that cerebral GLP‐1R activation effectively reduced TBI‐induced axonal injury and neuronal apoptosis.

Cognitive dysfunction is a common adverse consequence of TBI, which can reduce the patients' quality of life and affect their social reintegration.^35^ GLP‐1R activation in the brain improves cognitive performance in mice following TBI. However, when the glymphatic system was blocked beforehand, this improvement was not observed, suggesting that the glymphatic system could be the key pathway through which GLP‐1R exerts its neuroprotective effect. Owing to the complexity of the GLP‐1R pathway function, we focused on its possible side effects other than neuroprotection, such as hypoglycemia and weight loss, to ensure medication safety. GLP‐1R agonists stimulated insulin secretion in a glucose‐dependent manner. Thus, GLP‐1R agonists stimulate insulin secretion only in the cases of elevated blood glucose.^36^, ^37^ Our results demonstrate that Ex‐4 (10 μg/kg, administered intravenously), while exerting neuroprotective effects, does not cause significant disturbance to body weight and blood glucose.

The loss of perivascular AQP4 polarization following TBI contributed to the impairment of glymphatic pathway function.^5^, ^7^ AQP4 phosphorylation has been shown to play an important role in AQP4 trafficking and subcellular localization.^38^ Previous research shows that acute hypoxia leads to subcellular relocalization of AQP4 in primary cortical astrocytes. The transient receptor potential vanilloid 4 (TRPV4) channel, calmodulin (CaM), cyclic AMP (cAMP), and protein kinase A (PKA) were involved in the phosphorylation and subcellular relocation of AQP4.^39^ Therefore, CaM and PKA could be possible targets of cerebral GLP‐1R activation for glymphatic system regulation following TBI.

Our study has certain limitations. First, we administered a single intravenous injection to explore the neuroprotective effects against TBI. In future studies, subcutaneously embedded microosmotic pumps should be used for continuous administration to better simulate endogenous GLP‐1 secretion. Second, the molecular mechanism of GLP‐1R in the glymphatic system should be clarified in future studies.

## 5 CONCLUSIONS

This study suggests that cerebral GLP‐1R activation exerts a neuroprotective effect by ameliorating glymphatic system damage following TBI, thereby promoting recovery from TBI. Therefore, GLP‐1R could be a promising pharmacotherapeutic target for TBI.

## AUTHOR CONTRIBUTIONS

Chuanxiang Lv, Li Bie, and Rongcai Jiang conceived and designed the study. Chuanxiang Lv and Shuai Han wrote the manuscript, which was revised by Li Bie and Rongcai Jiang, approved by all the authors. Zhuang Sha, Mingqi Liu and Shiying Dong performed model preparation and CSF tracer infusions. Chunyun Zhang and Zean Li performed immunostaining. Kang Zhang and Shouyong Lu performed behavioral tests. Zhiyang Xu performed statistical analyses of the data.

## FUNDING INFORMATION

Science and Technology Development Plan Project of Jilin Province, China under Grant (20230204094YY) and (YDZJ202201ZYTS001).

## CONFLICT OF INTEREST STATEMENT

The authors declare that they have no competing interests.

## Supporting information


Appendix S1.

## Data Availability

The data that support the findings of this study are available from the corresponding author upon reasonable request.
