# Supplementary material for: Cerebral glucagon‐like peptide‐1 receptor activation alleviates traumatic brain injury by glymphatic system regulation in mice
Source: CNS Neurosci Ther. 2023 Jun 23;29(12):3876–88. doi: 10.1111/cns.14308 (PMC10651945; doi:10.1111/cns.14308)

Full unedited blot for Figure 3E

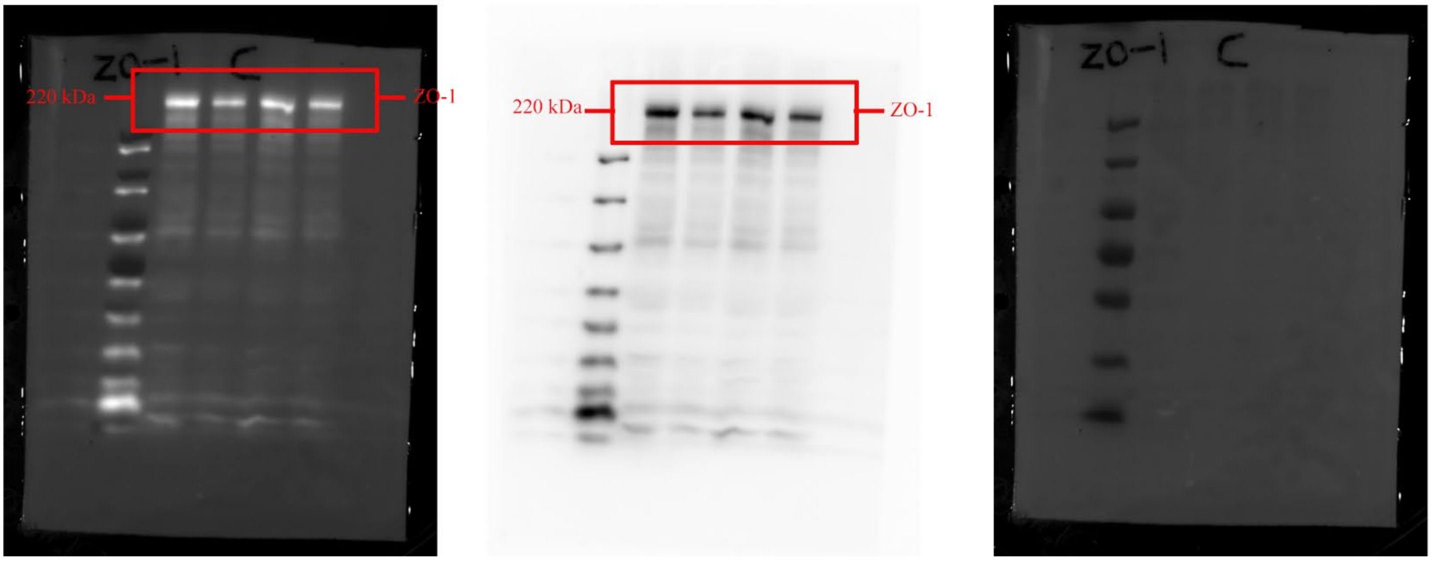

Full unedited blot for Figure 3E

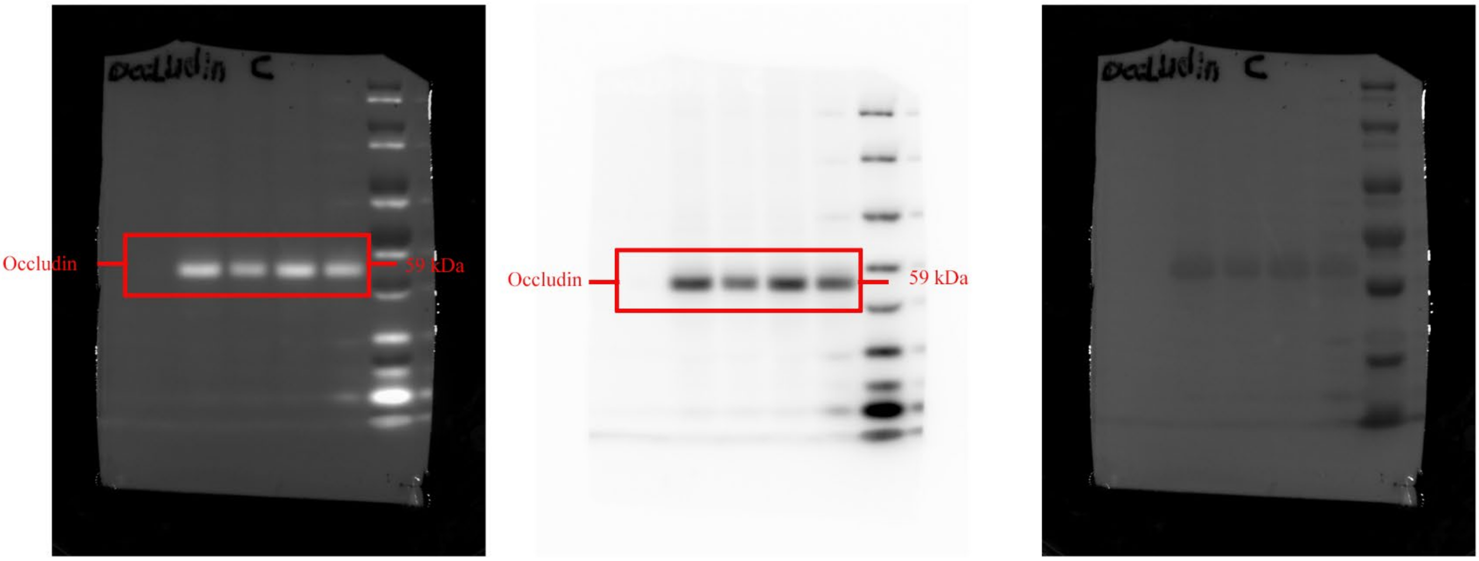

Full unedited blot for Figure 3E

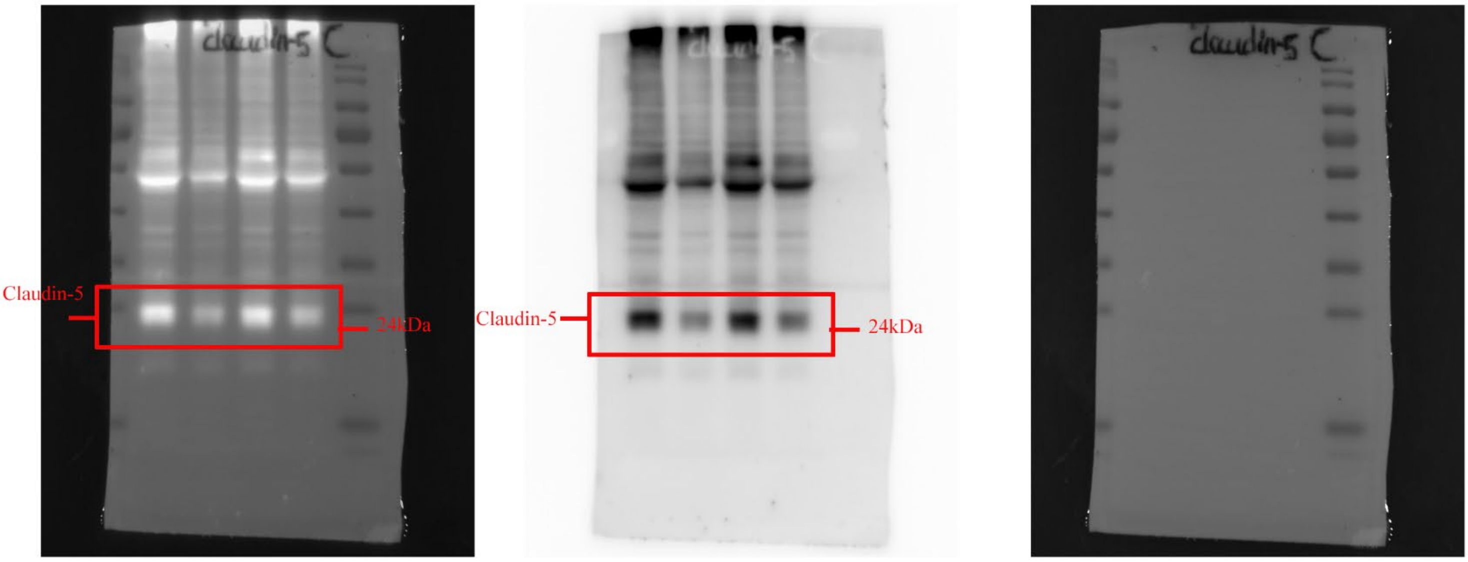

Full unedited blot for Figure 3E

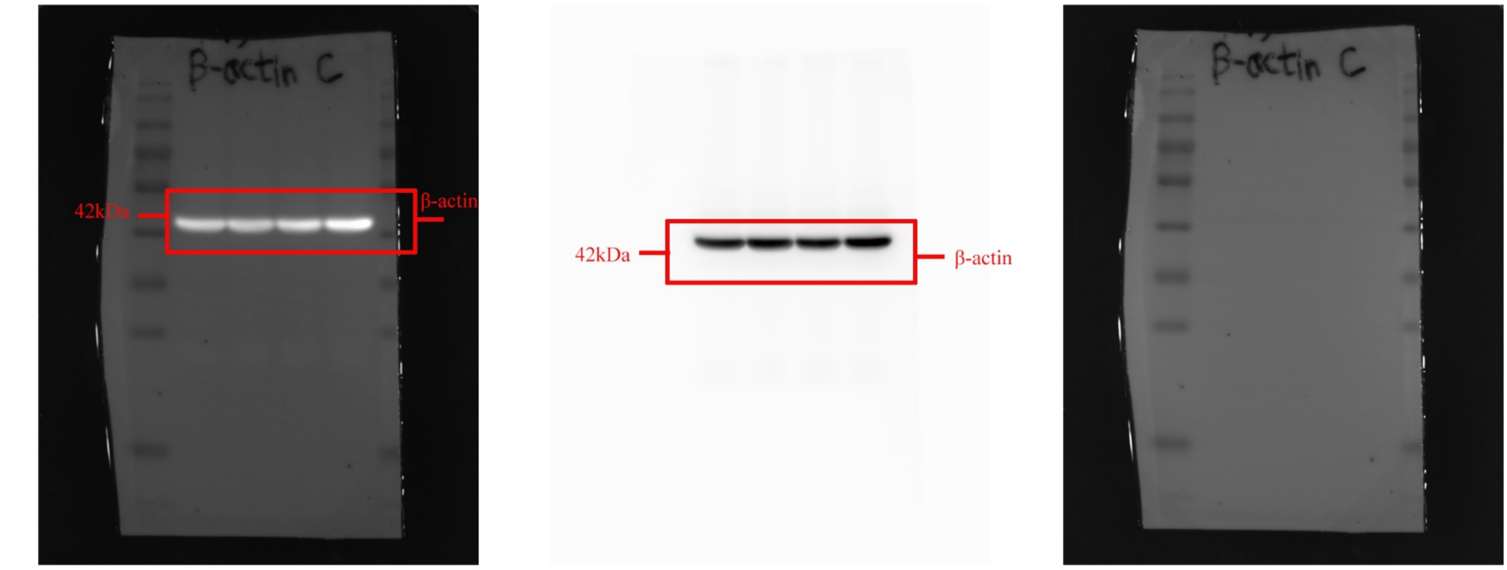

Full unedited blot for Figure 4F

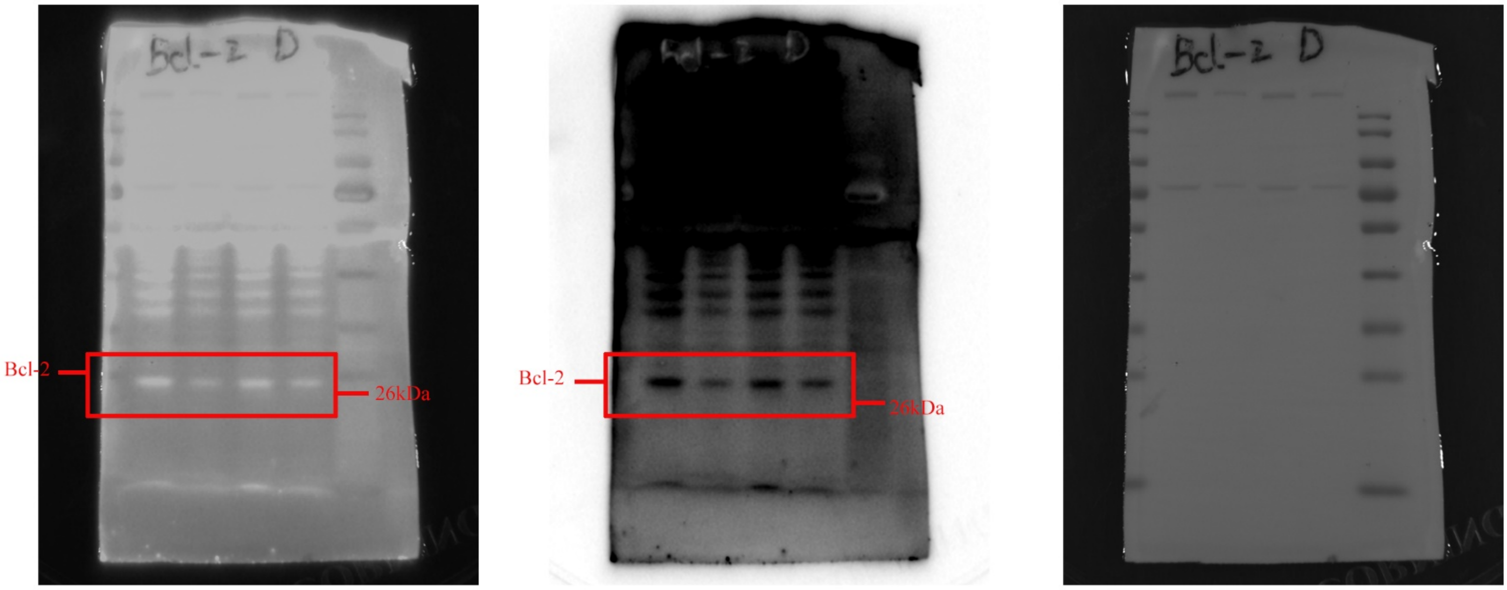

Full unedited blot for Figure 4F

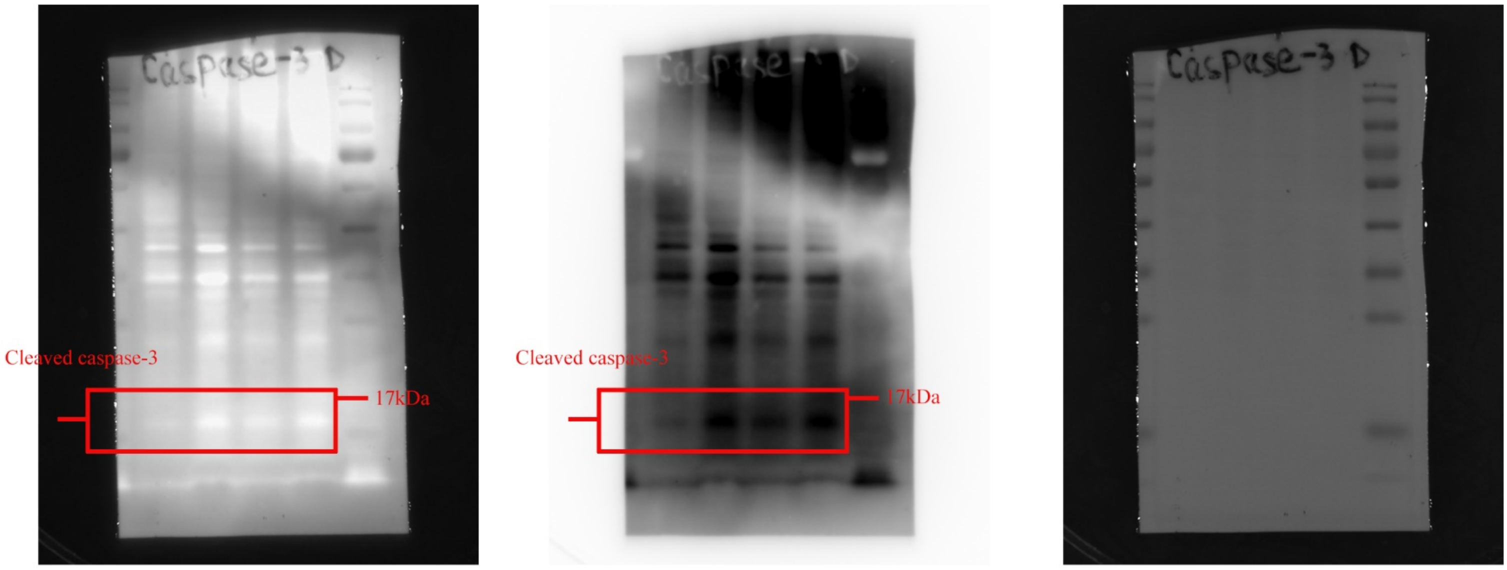

Full unedited blot for Figure 4F

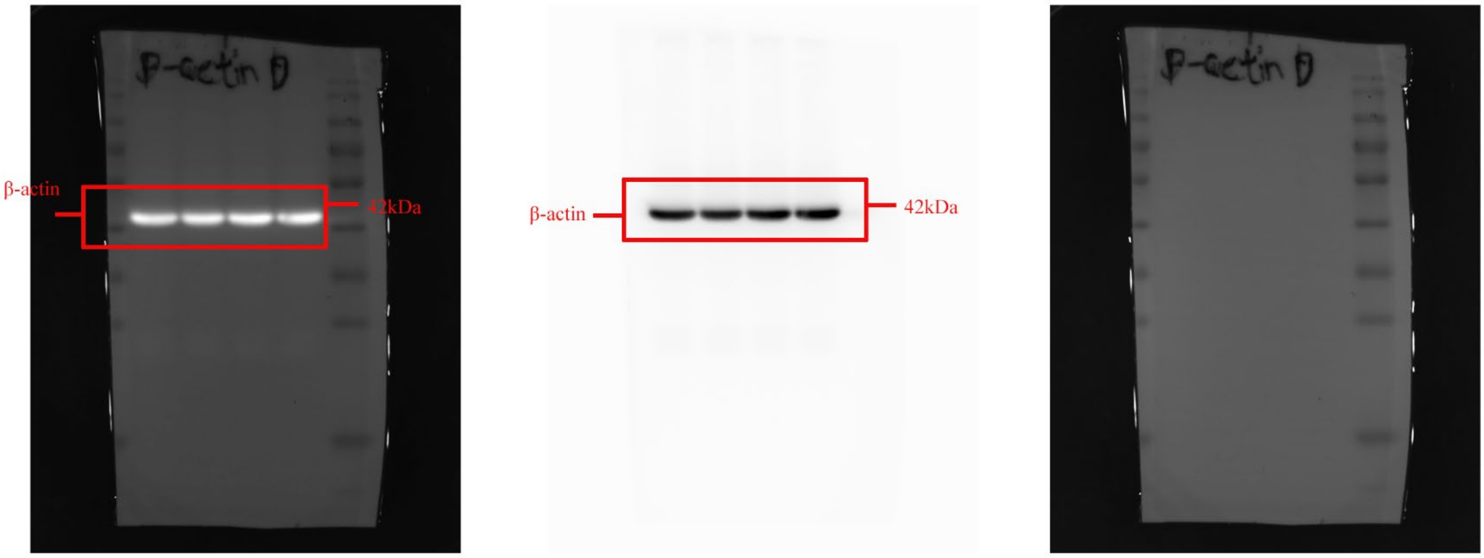

Supplement: Supplementary file 1 — Appendix S1. [file CNS-29-3876-s001.zip › Supplemental Files.pdf]
